# Shape effect of cerium oxide nanoparticles on mild traumatic brain injury

**DOI:** 10.1038/s41598-021-95057-9

**Published:** 2021-07-30

**Authors:** Dong Hyuk Youn, Ngoc Minh Tran, Bong Jun Kim, Youngmi Kim, Jin Pyeong Jeon, Hyojong Yoo

**Affiliations:** 1 Institute of New Frontier Research, Hallym University College of Medicine, Chuncheon, Republic of Korea; 2 Department of Materials Science and Chemical Engineering, Hanyang University, Ansan, Gyeonggi-do 15588 Republic of Korea; 3 Genetic and Research Inc., Chuncheon, Republic of Korea; 4 Department of Neurosurgery, Hallym University College of Medicine, 77 Sakju-ro, Chuncheon, 24253 Republic of Korea

**Keywords:** Neuroscience, Medical research, Neurology

## Abstract

The catalytic performance and therapeutic effect of nanoparticles varies with shape. Here, we investigated and compared the therapeutic outcomes of ceria nanospheres (Ceria NSs) and ceria nanorods (Ceria NRs) in an in vivo study of mild traumatic brain injury (mTBI). In vivo TBI was induced in a mouse model of open head injury using a stereotaxic impactor. Outcomes including cytoprotective effects, cognitive function, and cerebral edema were investigated after retro-orbital injection of 11.6 mM of ceria nanoparticles. Ceria nanoparticles significantly reduced fluoro-jade B (FJB)-positive cells and terminal deoxynucleotidyl transferase dUTP nick-end labeling (TUNEL)-positive cells, and restored mRNA levels of superoxide dismutase 1 (SOD1) and SOD2. They also decreased the cyclooxygenase-2 (COX-2) expression compared with the untreated control group. Comparing the two nanomaterials, Ceria NRs showed less stable and high-energy (100) and (110) planes, which increased the number of active sites. The Ce^3+^/Ce^4+^ molar ratio of Ceria NRs (0.40) was greater than that of Ceria NSs (0.27). Ceria NRs (0.059 ± 0.021) appeared to exhibit better anti-inflammatory effect than Ceria NSs (0.133 ± 0.024), but the effect was statistically insignificant (p = 0.190). Ceria nanoparticles also improved cognitive impairment following mTBI compared with the control group, but the effect did not differ significantly according to the nanoshape. However, Ceria NRs (70.1 ± 0.5%) significantly decreased brain water content compared with Ceria NSs (73.7 ± 0.4%; p = 0.0015), indicating a more effective reduction in brain edema (p = 0.0015). Compared with Ceria NSs, the Ceria NRs are more effective in alleviating cerebral edema following in vivo mTBI.

## Introduction

Traumatic brain injury (TBI) remains a major health concern resulting in death or disability. It accounts for a huge economic and health burden because the disease is relatively frequent at a young age. Approximately, 69 million individuals are estimated to sustain TBI each year^1^. In particular, the Southeast Asian and Western Pacific regions exhibit the greatest burden of TBI^1^. TBI can lead to neurologic sequelae via two different mechanisms including direct primary mechanical damage and secondary biochemical dysfunction involving acute to chronic phases^2,3^. Based on disease severity, TBI is categorized into mild, moderate and severe groups^4^. Generally, patients with mild TBI (mTBI) experience transient or no focal neurologic deficits including brief loss of consciousness without definite abnormalities based on initial radiological investigations such as computed tomography (CT) or magnetic resonance imaging (MRI)^4^. However, approximately 10% to 15% of patients with mTBI may continue to suffer from memory impairment with decreased attention and awareness, which is a major challenge clinically^5,6^. Although minimal or no cell death occurs from primary brain injury in such cases, secondary brain injury due to cellular dysfunction due to imbalance between increased oxidative stress and endogenous antioxidants and radical scavengers can lead to persistent cellular injury and cognitive dysfunction eventually^7–9^. Antioxidant drugs have been investigated in clinical studies to ameliorate oxidative damage, although effective drug treatment has yet to be reported^7^.

Cerium oxides (ceria) have attracted substantial attention in the field of environmental applications and catalysis^10^. Ceria nanoparticles have demonstrated satisfactory antioxidant, antibacterial, and anticancer functions as well as high resistance to cytotoxicity and neurotoxicity^11,12^. The widespread use of ceria nanoparticles is attributed to their high catalytic activities owing to rapid transformation of the oxidative states of Ce^3+^ and Ce^4+^ (so called oxygen storage capacity)^13^. In addition, ceria nanoparticles exhibit oxygen vacancies in the lattice structure due to the loss of electrons or oxygen atoms, leading to a switch between CeO_2_ and CeO_2-x_ during redox processes^14^. Controlling the morphology is critical to the catalytic performance of ceria nanoparticles, since the selective exposure of reactive crystal planes on the surface can enrich the catalytic sites^15,16^. Recent studies reported that ceria nanoparticles with a spherical shape decreased neuronal cell death and calcium dysregulation by preserving the antioxidant system in the mTBI model^7^. However, no comparison with other nanodrugs was made, and the studies merely demonstrated better therapeutic efficacy of ceria nanoparticle in injured mice compared with untreated animals^7^. Therefore, we compared the possible differences in therapeutic effects of ceria nanoparticles (nanospheres vs. nanorods) in a mouse model of mTBI.

## Results

### Characterization of ceria nanoparticles

**Figure 1:**
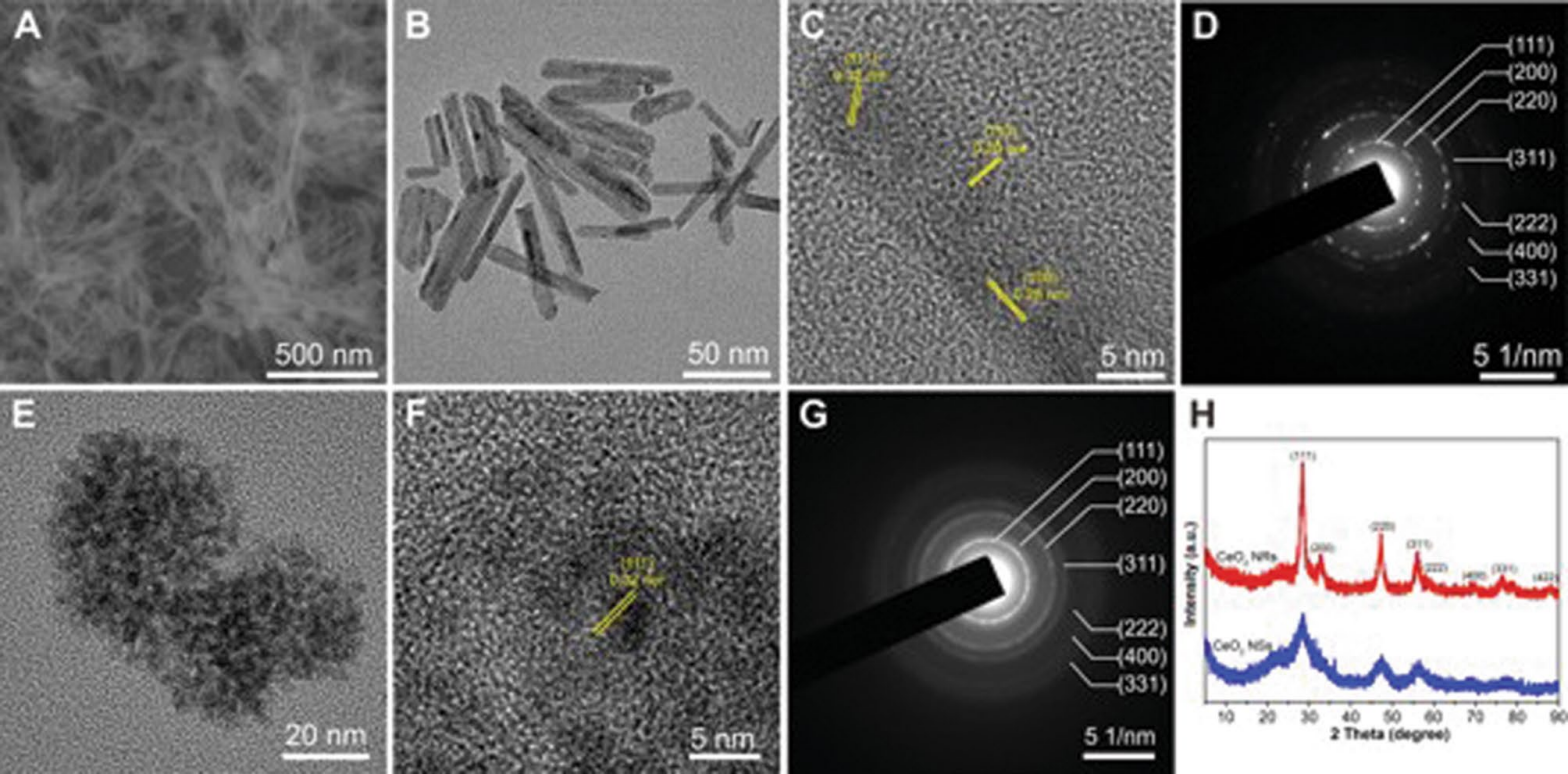
(**A**). SEM image of Ceria (CeO_2_) NRs. (**B**, **E**) TEM images, (**C**, **F**) High-resolution TEM images, and (**D**, **G**) SAED patterns of Ceria NRs and Ceria NSs, respectively; and (**H**) PXRD patterns of Ceria NRs (in red) and Ceria NSs (in blue).

**Figure 2:**
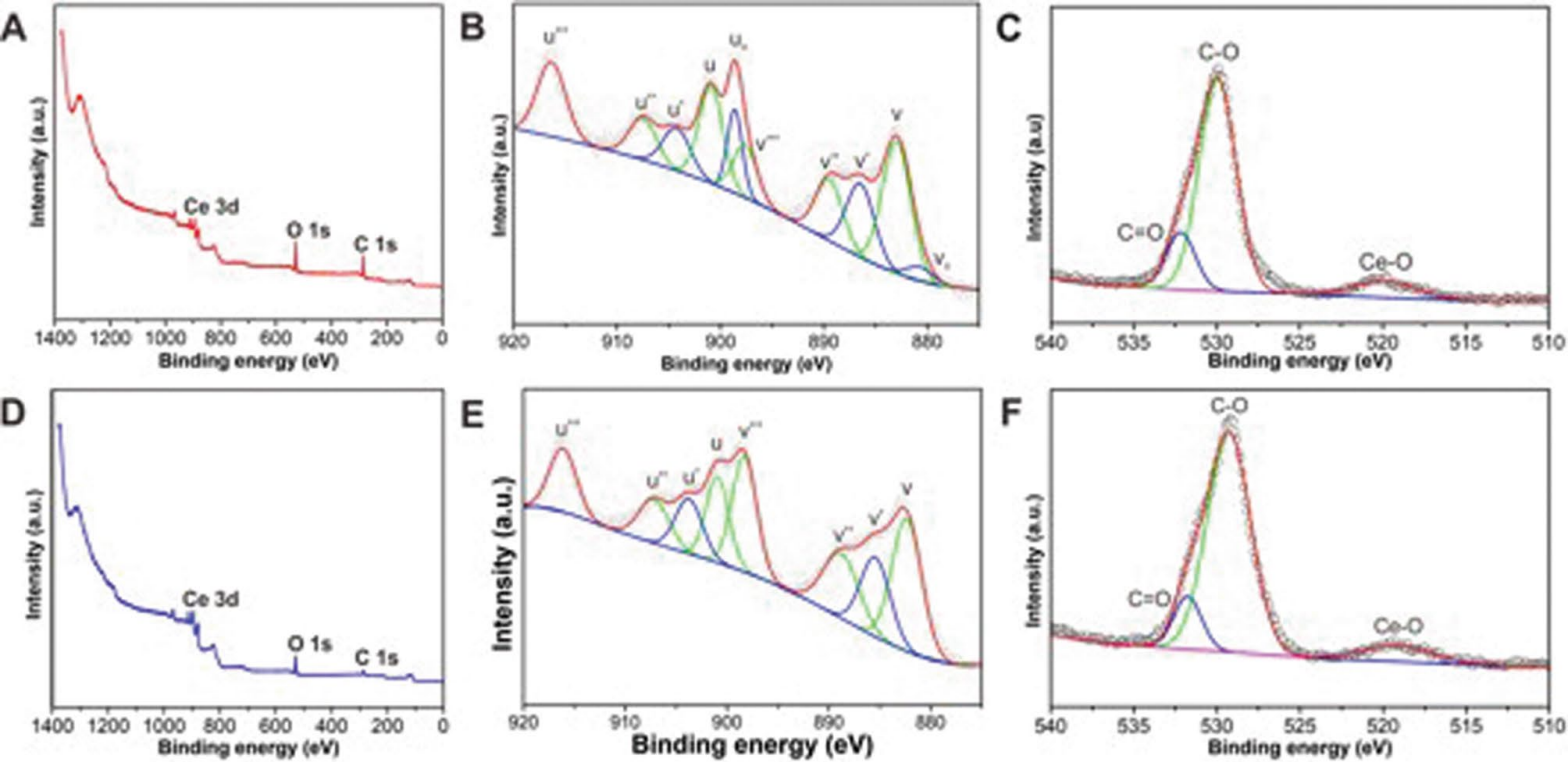
XPS survey spectra (**A**, **D**), XPS high-resolution binding energy spectra of Ce 3*d* (**B**, **E**) and O 1*s* (**C**, **F**) of Ceria NRs and Ceria NSs, respectively. Ce^4+^ and Ce^3+^ were coexistence in Ceria NRs and Ceria NSs.

Scanning electron microscope (SEM) and transmission electron microscope (TEM) images (Fig. 1A,B) of the obtained products revealed that Ceria nanorods (Ceria NRs) had a rod-like morphology measuring 130.1 ± 42.1 (nm) in length and 9.4 ± 2.1 (nm) in diameter (Fig. S1 A and B), while Ceria nanospheres (Ceria NSs) (Fig. 1E) were uniform and spherical with a mean particle size of approximately 3.5 ± 0.5 nm (Fig. S1C). High-resolution TEM images (Fig. 1C,F) revealed the exposed crystal planes on the surface of Ceria NRs and Ceria NSs, demonstrating the highly crystalline nature of the resulting ceria nanoparticles. Ceria NRs contained exposed (100), (110), and (111) planes, whereas only (111) plane was exposed on the surface of Ceria NSs. The selected area electron diffraction (SAED) (Fig. 1D,G) and powder X-ray diffraction (PXRD) patterns (Fig. 1H) of the two ceria nanoparticles were similar. The predominant peaks were indexed to (111), (200), (220), (311), (222), (400), and (311) planes indicated that both ceria nanoparticles were assigned to the pure fluorite cubic structures of CeO_2_ (JCPDS 34-0394, space group *Fm-3m*). No diffraction peaks belonging to other foreign components were detected, indicating the purity of the obtained products. The composition of the ceria nanoparticles was further established using EDX elemental mapping data (Fig. S2). The specific surface area (S_BET_) of Ceria NRs and Ceria NSs were 76 and 230 m^2^ g^−1^, respectively (Fig. S3). The characterization of ceria nanoparticles were summarized in Table 1. In contrast to the white color of pure CeO_2_, the resulted ceria nanoparticles exhibited a yellowish color, which demonstrated the coexistence of not only Ce^4+^ but also Ce^3+^ in the structure. X-ray photoelectron spectroscopy (XPS) analysis revealed a mixed valence state of Ce^4+^ and Ce^3+^ (Fig. 2). Figure 2A and D showed the XPS survey spectra of Ceria NRs and Ceria NSs. Ceria NRs exhibited characteristic peaks of Ce^4+^ with binding energy of 882.9, 889.4, 897.6, 900.9, 907.5, and 916.3 eV, while the peaks at the binding energy of 880.7, 886.4, 898.6, and 904.2 eV corresponded to Ce^3+^ (Fig. 2B). In the case of Ceria NSs (Fig. 2E), the peaks were observed at the binding energy of 882.3, 888.7, 898.2, 900.9, 907.1, 916.1 eV in Ce^4+^ and 885.4, 903.7 eV in Ce^3+^**.** The molar ratio between fitted peak areas of Ce^4+^ and Ce^3+^ was used to estimate their concentrations on the surface of ceria nanoparticles. As shown in Table S1, the Ce^3+^/Ce^4+^ molar ratio of Ceria NRs and Ceria NSs are 0.40 and 0.27, respectively. This data indicate that Ceria NRs contained more Ce^3+^ on the surface than Ceria NSs. It is well-known that the biomedical activity of ceria nanoparticles is enhanced by higher levels of Ce^3+^ on the surface. As shown in Fig. 2C and F, in the XPS high-resolution binding energy spectra of O 1 s of Ceria NRs and Ceria NSs the first peak at around 529 eV was associated with lattice oxygen. A higher binding energy shoulder at around 532 eV was assigned to oxygen vacancies or a mixture of surface adsorbed oxygen, hydroxyl, and carbonate groups.

**Table 1:** Characterization of ceria nanoparticles.

Particle characteristic	Ceria NRs	Ceria NSs
Morphology	Nanorod	Nanosphere
Size (nm)	Length: 130.1 ± 42.1 Diameter: 9.4 ± 2.1	Diameter: 3.5 ± 0.5
Surface Ce^3+^/Ce^4+^ ratio	0.40	0.27
S_BET_ (m^2^ g^−1^)	76	230

*S*_*BET*_ Brunauer–Emmett–Teller (BET) surface area of ceria nanoparticles.

### Cytoprotective effect in in vivo mild TBI

**Figure 3:**
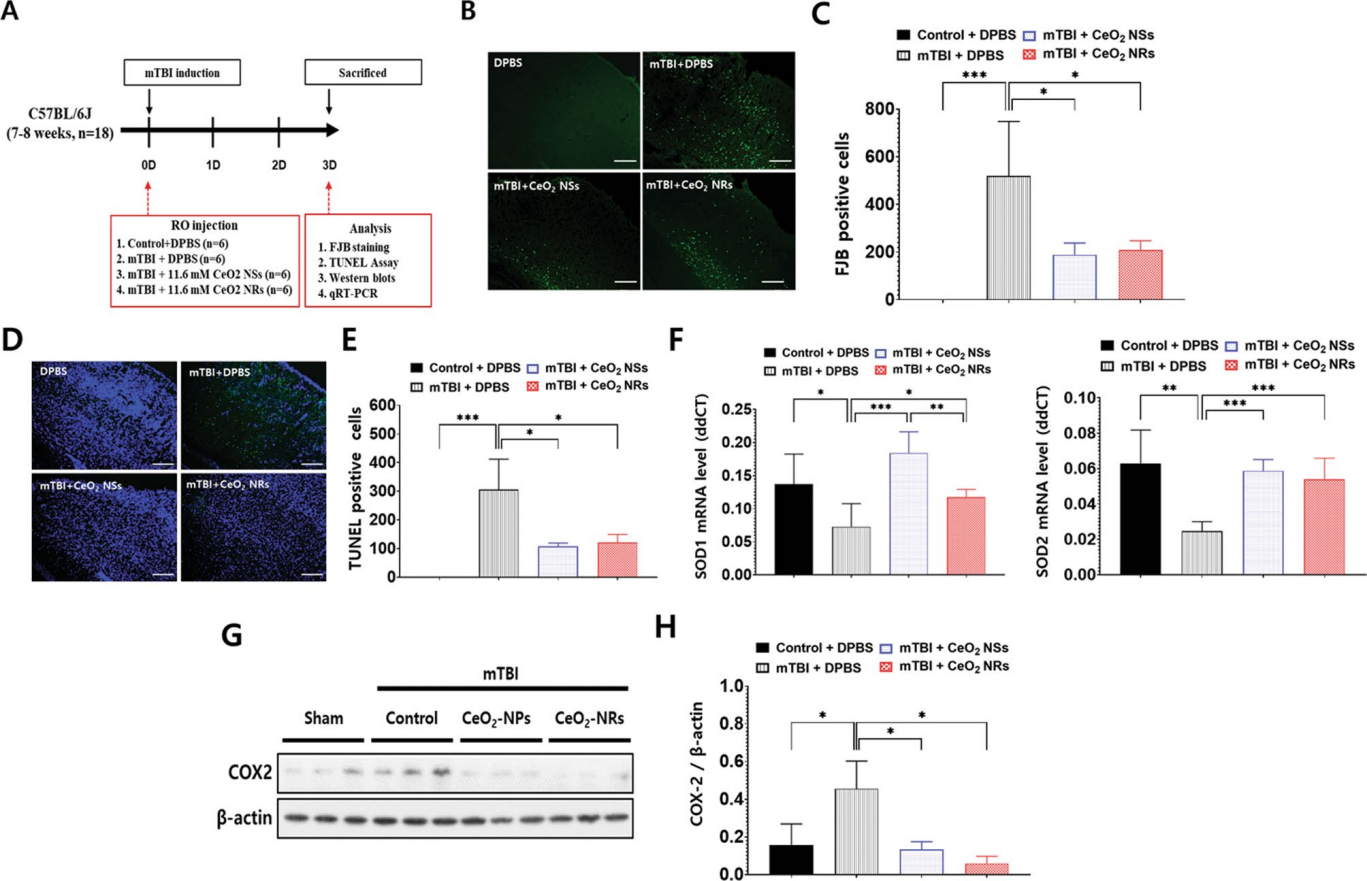
Schematic diagram of in vivo study (**A**). Fluoro-Jade B (FJB) staining was used to detect the neuronal death (**B**, **C**). Fluorescence images represent the relative degree of neuronal death in the cortex. The number of FJB-positive cells were decreased in Ceria NRs and Ceria NSs than in DBPS group. TUNEL assay in mTBI in vivo (**D**, **E**). Representative images of TUNEL-positive green cells and its quantification. DAPI was used to counterstaining (blue). The level of SOD1 and SOD2 mRNA expression was analyzed using qRT-PCR (**F**). Western blot analysis of COX-2 expression (**G**) and quantification of blots is based on relative optical densities of COX-2 and β-actin protein (**H**). Scale bar = 200 μm. Error bars, mean SEM, *P < 0.05, ** P < 0.01, and ***P < 0.001.

Fluoro-Jade B (FJB) staining and terminal deoxynucleotidyl transferase dUTP nick end labeling (TUNEL) assay were performed to further evaluate the role of nanoparticle shape in the anti-inflammatory activity and subsequent protection of neuronal cell death in mTBI in vivo. As shown in the schematic diagram (Fig. 3A), mice were sacrificed 3 days after mTBI with or without injection with Ceria NRs or Ceria NSs. No FJB-positive cells were detected in the control group. FJB-positive neuronal cell injury was observed in the cortex area after mTBI (Fig. 3B). Compared with DPBS-treated mTBI group (519.552 ± 76.185), the ceria nanoparticle-injected (11.6 mM/mice) group showed a significant reduction in the number of FJB-positive cells (188.981 ± 16.329 in the Ceria NSs and 209.205 ± 12.785 in the Ceria NRs) (Fig. 3B,C). Consistent with FJB staining, both Ceria NRs and Ceria NSs significantly decreased the number of TUNEL-positive cells compared to the mTBI group (Fig. 3D,E). Treatment with ceria nanoparticles restored the decreased mRNA levels of SOD1 and SOD2 following mTBI (Fig. 3F). Furthermore, COX-2 expression was also remarkably reduced in the groups treated with ceria nanoparticles compared with the control group (Fig. 3G,H, and Supplemental Fig. S4). These results suggest the cytoprotective effects of ceria nanoparticles via suppression of inflammation and oxidative stress in injured neuronal cells, but the anti-oxidative activities did not differ significantly according to the nanoshape. Ceria NRs (0.059 ± 0.021) appeared to exhibit better anti-inflammatory effect than Ceria NSs (0.133 ± 0.024), although the effects were statistically insignificant (p = 0.190).

### Cognitive function in in vivo mild TBI

**Figure 4:**
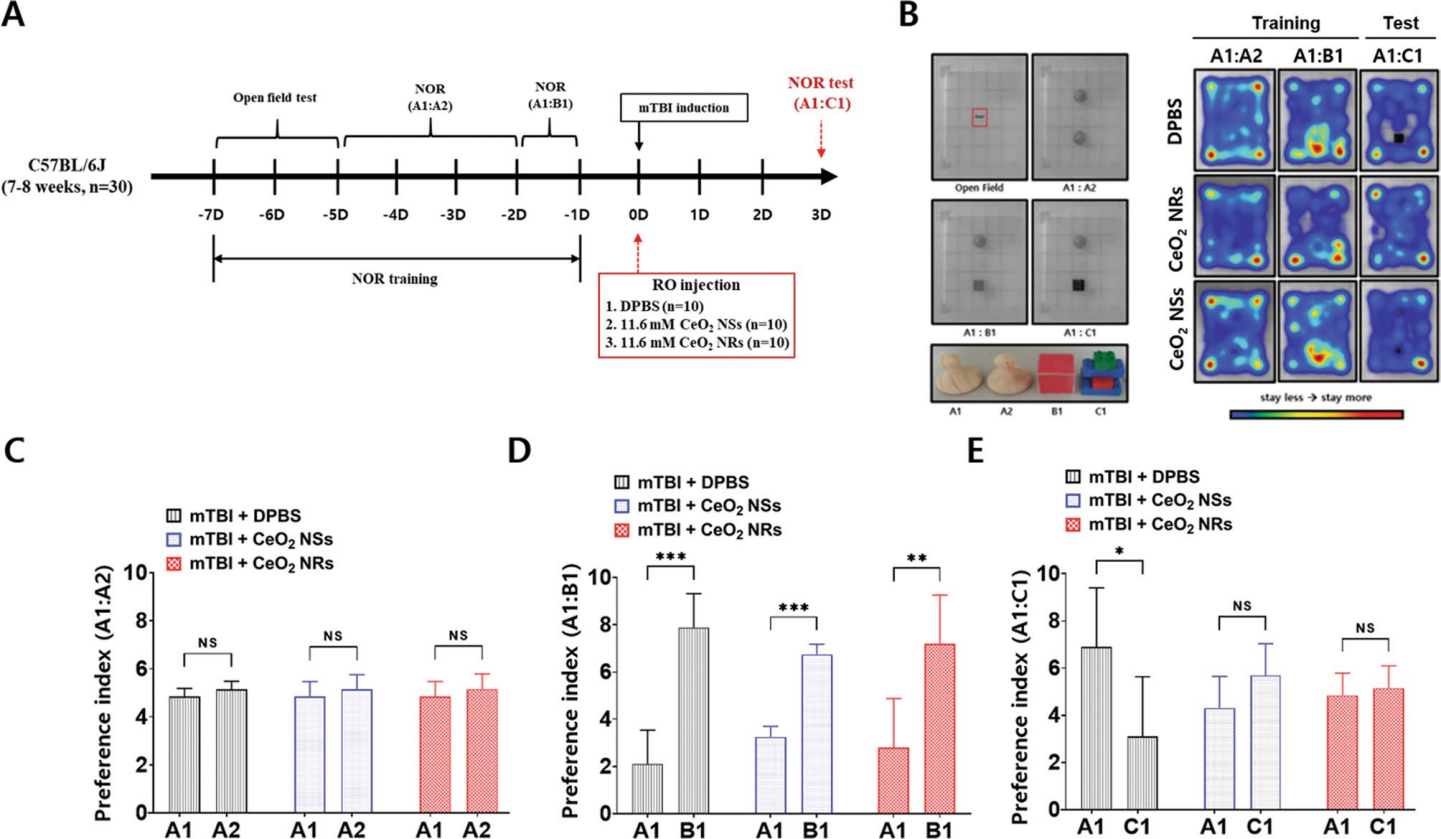
Experimental design of the Novel Object Recognition (NOR) test (**A**). Altered object exploration ration in the NOR test. Heat map analysis of animal tracking following NOR test (**B**). Each group shows similar preferential investigation of the novel objects before mTBI (**C**, **D**). Differences in the degree of impaired discrimination index following mTBI between DPBS and Ceria NSs or Ceria NRs (**E**). Mice treated with cerium oxide nanoparticles showed better preference for novel objects than DPBS, indicating cognitive improvement after injury.

A schematic representation of in vivo study of novel object recognition (NOR) and its explanation are presented in Fig. 4 and Supplemental data, respectively. The average preference index for each group in Fig. 4B (right) is summarized in Figs. 4C–E. Each groups showed intact NOR memory before mTBI (Fig. 4C,D). Following mTBI, mice showed memory impairment (Fig. 4B, right upper) and the corresponding preference index was approximately equal to 3.0 (Fig. 4E, gray bar). Both ceria nanoparticles improved NOR memory in mTBI-induced mice (Fig. 4E; preference index for the novel object of C1, shown in blue and red bars). Thus, ceria nanoparticles improved cognitive function, but the effect was not different significantly according to the shapes of NRs and NSs.

### Cerebral edema in in vivo mild TBI

**Figure 5:**
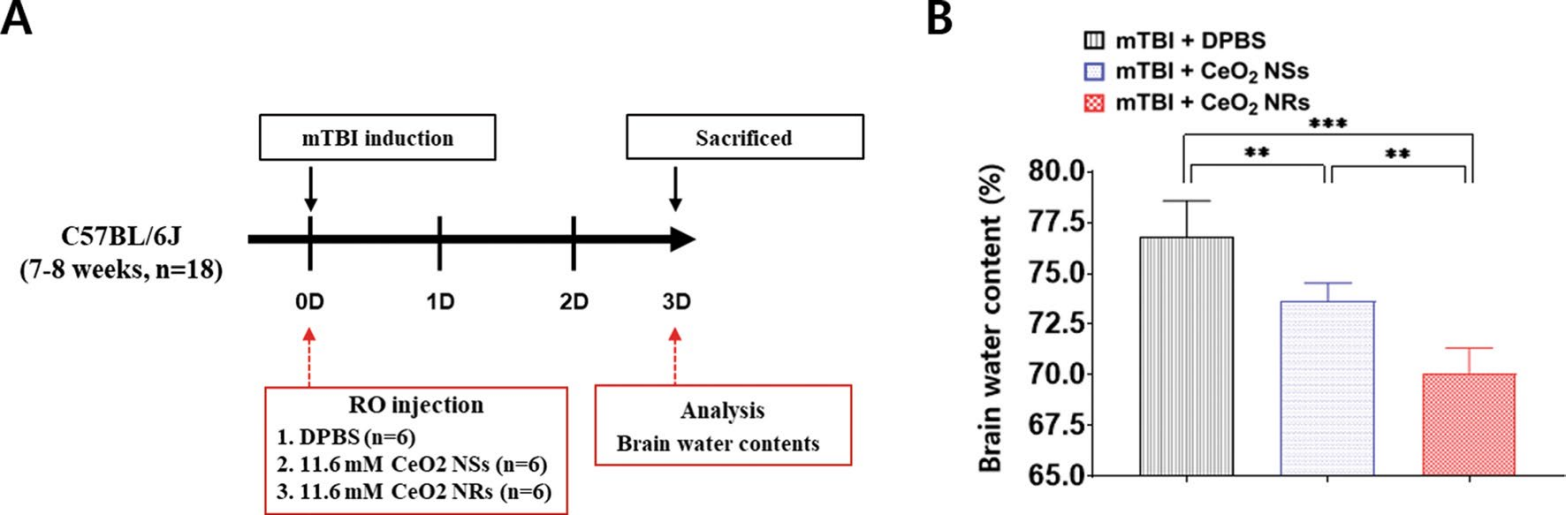
Schematic diagram depicts brain water content analysis of the degree of cerebral edema (**A**). Comparison of brain water content in the three groups: DPBS, Ceria NSs, and Ceria NRs. Error bars, mean SEM, **P < 0.01, and ***P < 0.001.

The cerebral edema was compared additionally via estimation of changes in overall water content of the brain tissues as described previously^17^. A schematic diagram of the measurement of cerebral edema is provided in Fig. 5. Ceria nanoparticles significantly decreased the cerebral edema than in the control group (DPBS-treated mTBI group). Compared with Ceria NSs (73.7 ± 0.4%), Ceria NRs (70.1 ± 0.5%) significantly decreased brain water content, demonstrating a more effective decline in brain edema (P = 0.0015).

## Discussion

Neuroprotective effects of ceria nanoparticles have been mainly investigated in neurodegenerative and cerebrovascular diseases associated with defective antioxidant mechanisms and subsequent susceptibility to oxidative stress^18^. Dowding et al.^19^ reported that ceria nanoparticles reduced the levels of Aβ-induced mitochondrial fragmentation, endogenous peroxynitrite and neuronal cell death. Treatment with 10 nM ceria nanoparticles provided confluent astrocyte monolayers with multiple processed neurons^20^. In addition, cultures treated with ceria nanoparticles exhibited higher catalase levels and decreased glutathione compared with untreated controls during the life span^20^. Treatment with ceria nanoparticles at doses ranging from 0.5 to 0.7 mg kg^−1^, also reduced the infarct volume following acute ischemic stroke^21^. However, only a few studies have focused on the efficacy of ceria nanoparticles in TBI, although excessive oxidative stress is a major damage mechanism in the brain after TBI^22^. In rat models with moderate TBI, pretreatment with ceria nanoparticles showed improved task performance and recovery than in control groups^22^. Bailey et al.^7^ investigated treatment outcomes of ceria nanoparticles in rats with mild lateral fluid percussion brain injury and showed that ceria nanoparticles significantly improved catalase activity in the injured brain. During the cellular uptake of ceria nanoparticles, the shape can affect endocytosis and determine the efficacy of these agents. The shape effect of nanoparticles is mainly determined by different membrane-bending energies during endocytosis^23^. Spherical ceria nanoparticles required a minimal membrane-bending energy barrier than the non-spherical counterparts. Li et al.^23^ concluded that the shape of the nanomaterial only affects the free energy change of grafted polyethylene glycol (PEG) polymers during internalization.

In brief, we first tested the ability of ceria nanoparticles based on their shape in an in vivo mTBI model. Theoretically, Ceria NRs exhibit better antioxidant activity and cytotoxicity than Ceria NSs for the following reasons. First, the difference in reactive crystal planes is attributed to the difference in therapeutic effects. As shown by high-resolution TEM analysis (Fig. 1), the less stable and high-energy (100) and (110) planes were predominantly exposed in Ceria NRs. However, the (111) plane on the surface was mainly exposed in Ceria NSs, which is consistent with previous reports^24^. It is believed that the improved biomedical activities of Ceria NRs originate in the abundance of (100) and (110) terminated planes compared with Ceria NSs, since the crystalline planes with high surface energy provide more active sites. The effects of structural features in ceria nanoparticles on the catalysis such as CO oxidation have been investigated comprehensively^16,25^. For example, Ceria NRs were found to be more active in CO oxidation than nanocubes, nanowires, and nanoparticles^16,26^. Similarly, Co_3_O_4_ NR with a higher exposure of the reactive crystal planes also exhibited particularly high activity towards CO oxidation at low temperature^27^. Second, the Ce^3+^/Ce^4+^ molar ratio on the surface of ceria nanoparticles also affects the biomedical activities^28,29^. By changing the oxidation state from Ce^3+^ to Ce^4+^, ceria nanoparticles can scavenge the free radicals or reactive oxygen species^11^. Meanwhile, changing the oxidation state from Ce^4+^ to Ce^3+^ in ceria nanoparticles completes the autoregenerative reaction cycle^30^. As shown in Fig. 5, the Ce^3+^/Ce^4+^ molar ratio of Ceria NRs (0.40) is greater than that of Ceria NSs (0.27) (calculated from the XPS data), which indicates the concentration of Ce^3+^ on the surface of Ceria NRs is higher than Ceria NSs. Third, the oxygen vacancies in the lattice structure of ceria nanoparticles, which are the most active on the surface, also play an essential role in determining their biomedical properties. Alteration in the oxidative state of ceria nanoparticles generates oxygen vacancies to maintain electrostatic balance via loss of oxygen and electrons^24^. The increased number of Ce^3+^ sites in the ceria nanoparticles increases the number of oxygen vacancies on the surface. Nevertheless, in our study, anti-oxidant and anti-inflammation activities in mTBI in vivo did not differ significantly according to nanoshape, although the mRNA expressions of SOD1 and SOD2, and COX-2 tended to decrease in Ceria NRs than Ceria NSs. However, cerebral edema was significantly reduced in Ceria NRs compared with Ceria NSs following mTBI. In addition, there was no statistically significant difference in anti-inflammatory effect and improvement in cognitive dysfunction according to the shape of nanoparticles. We speculated that the relatively high dose of nanoparticles and the mild TBI severity may contribute to these outcomes. In this study, we used a dose of 11.6 mM based on a literature review, which was higher than the dose used in other studies^21,31^. Additionally, we only targeted mild TBI, not moderate-to-severe TBI. Thus, there was a possibility that the relationship between improvement in anti-inflammatory effect and cognitive dysfunction and nano-shape was unclear. Therefore, studies investigating the improvement of cognitive function according to nano-shape are needed based on various dosages of nanoparticles in different degrees of TBI injury.

Shape of nanoparticles can influence biological effects as well as their size and surface charge^32^. Zhao et al.^32^ reported that long rods showed longer circulation in the blood with less rapid clearance by reticuloendothelial system. Rods were also degraded faster than spherical nanoparticles due to their higher specific surface area. In addition, there may be less aggregation among rod nanoparticles than the spherical particles and thus expected to be less toxic due to the small dosage of rods with high oxygen vacancies on the surface than the spherical nanoparticles. It is believed that ceria substances are chemically stable and not significantly affected by chemical changes during long-term treatment. These findings suggested that Ceria NRs may have a higher bioavailability than Ceria NSs. The use of Ceria NRs in clinical practice requires a further study investigating the therapeutic efficacy based on rod length and effective nano drug delivery methods without loss of activity during indigestion. Although results of TBI according to gender difference vary and are model-dependent^33^, many studies reported that females may manifest neuroprotective effects following TBI^34^. Shahrokhi et al.^35^ reported that estrogen and progesterone exhibited decreased intracranial pressure and improved cerebral perfusion pressure after TBI in ovariectomized rats, suggesting that sex hormones may be neuroprotective following TBI. This study was a proof-of-concept study that focused on identifying the influence of ceria shape on outcome in mTBI. Thus, male mice were only used to eliminate the confounding variable of TBI outcome based on potential gender differences. Nevertheless, application in real-world clinical and drug development requires research into the therapeutic effects based on toxicity and appropriate dosage according to gender.

The study limitations are as follows. First, cerebral edema was estimated using the water content based on the difference between wet and dry weights^17^. In such cases, small changes in the percentage of water content reflect the relatively large changes in water component of the tissue^17,36^. Second, long-term outcomes of the ceria nanoparticles according to shape were not investigated. Ceria NRs showed similar anti-oxidant and anti-inflammation activities than Ceria NSs, but the effect was usually limited to the acute phase after mTBI. Third, cellular toxicity and clearance according to changes in nanoshape were not tested before clinical application in patient with mTBI.

## Conclusions

Ceria nanoparticles decreased neuronal damages and improved cognitive impairment in vivo mTBI. Compared to Ceria NSs, Ceria NRs demonstrated better effects on reduction of cerebral edema.

## Materials and methods

### Synthesis of ceria nanoparticles

Two types of ceria nanoparticles were synthesized. Ceria NRs were prepared by modifying the previously reported protocol^37^. Cerium(III) nitrate hexahydrate (Ce(NO_3_)_3_·6H_2_O, 99%, Sigma-Aldrich) and sodium hydroxide (NaOH, 98%, Sigma-Aldrich) were used. Typically, Ce(NO_3_)_3_·6H_2_O (0.434 g, 1 mmol) was added to an aqueous solution of NaOH (10 M, 20 mL) in a PTFE beaker. The reaction mixture was vigorously stirred for 2 h at room temperature before transferring to a teflon-lined stainless-steel autoclave and was incubated in a temperature-controlled oven at 100 °C for 10 h. The mixture was naturally cooled to room temperature after completion of the reaction. The reaction product was collected by centrifugation, washed several times with solvents (deionized water and ethanol), and then vacuum-dried for 24 h for further use. Ceria NSs were synthesized using a reported protocol with slight modification^38^. Cerium(III) nitrate hexahydrate (Ce(NO_3_)_3_·6H_2_O, 99%, Sigma-Aldrich), oleylamine (C_18_H_37_N, 70%, Sigma-Aldrich), and xylene (C_8_H_10_, 98.5%, Junsei) were used. Typically, Ce(NO_3_)_3_·6H_2_O (0.434 g, 1 mmol) and C_18_H_37_N (3.25 g, 12 mmol) were dissolved in C_8_H_10_ (15 mL). The resulting solution was vigorously stirred for 2 h at 25 °C and then heated to 90 °C at the rate of 2 °C.min^-1^ under vacuum. Deionized water (1 mL) was rapidly injected into the solution under vigorous stirring at 90 °C to initiate the sol–gel reaction, as indicated by a color change from purple to cloudy yellow. The reaction mixture was incubated at 90 °C for 3 h to obtain a light-yellow colloidal solution. The mixture was cooled to ambient temperature, and Ceria NSs were precipitated by adding ethanol (75 mL). The product was centrifuged and washed several times with ethanol, and then dried under vacuum for 24 h for further use.

### In vivo mild TBI

All animal experiments were approved by Institutional Animal Care and Use Committee (IACUC) of Hallym University (approval no. HallymR2 2019-35). All animal study protocol was carried out according to the ARRIVE guidelines (Animal Research: Reporting of In Vivo Experiments). VC57BL/6J male mice, 7–8 weeks of age, were obtained from the Laboratory Animal Resources Center, Hallym University, Korea. The animal was provided with regular food and water ad libitium under a 12 h dark/light cycle at 24 °C, 55 ± 10% humidity. The different experimental groups were as follows: (1) mTBI (n = 16), (2) mTBI treated with Ceria NRs (n = 16), (3) mTBI treated with Ceria NSs (n = 16), and sham operation (n = 6).

In vivo TBI was induced via open head injury using a stereotaxic impactor (RWD Life Science, RWD-68099, China)^39^. In brief, the C57BL/6J male mice were anesthetized using 2.5% isofurane in oxygen and placed in the stereotaxic frame. The skull was exposed via a midline skin incision, and TBI was induced as follows: M/L = – 2.5 mm, A/P = − 2.0 mm, from bregma, at 1.5 mm depth using a blant tip with a diameter of 2 mm. The velocity of the impactor reached 3.0 m/s with a depth of 1.5 mm using the 2 mm blant tip below the dura matter. The dwell time in the brain was 0.5 m/s. Ceria nanoparticles accumulate in the brain via a bell-shaped dose–response curve^7,40^, and therefore, a single retro-orbital injection with a dose of 11.6 mM of ceria nanoparticles was administered in this study^41^. The various assay such as FJB, TUNEL, western blot, measurement of cerebral edema^17,36^, and behavioral tests including NOR are described in the Supplemental Methods. Outcome measurements were carried out by investigators who were blinded to the treatment methods of mice. The study protocol is performed in accordance with the relevant guidelines.

### Statistical analysis

All data were presented as the means ± standard errors of the mean (SEM). Student’s t-test or one-way ANOVA with post-hoc Bonferroni correction was conducted for all possible pair-wise comparisons^21^. *P* value less than of < 0.05, 0.01, and 0.001 are represented by *, **, and *** in the figures, respectively^42^. All statistical analyses were performed with GraphPad Prism software (v.6.01; GraphPad Software Inc., San Diego, CA, USA).

## Supplementary Information

Supplementary Information.

## Data Availability

The data support the findings of this study are available from the corresponding author upon reasonable request.
