# Supplementary material for: Shape effect of cerium oxide nanoparticles on mild traumatic brain injury
Source: Sci Rep. 2021 Jul 30;11:15571. doi: 10.1038/s41598-021-95057-9 (PMC8324865; doi:10.1038/s41598-021-95057-9)
Supplement: Supplementary file 1 — Supplementary Information. [file 41598_2021_95057_MOESM1_ESM.docx]

**Supplemental data**

**Table S1.** Surface composition of ceria nanoparticle.

| **Sample** | **Surface percentage (%)** | | | **Ce^3+^/ Ce^4+^ ratio*** |
| --- | --- | --- | --- | --- |
|  | Ce 3d  (Total) | Ce 3d  (Ce^3+^) | Ce 3d  (Ce^4+^) |  |
| Ceria NRs | 100 | 28.40 | 71.60 | 0.40 |
| Ceria NSs | 100 | 21.28 | 78.72 | 0.27 |

Ceria NSs, ceria nanospheres; Ceria NRs, ceria nanorods.

*Ce^3+^/ Ce^4+^ ratio is calculated using the following formula:

For Ceria NRs:

$$\left[ {Ce}^{3+} \right]=\frac{A_{v_{o}}+A_{v^{'}}+A_{u_{o}}+A_{u^{'}}}{A_{v_{o}}+A_{v^{'}}+A_{u_{o}}+A_{u^{'}}+A_{v}+A_{v^{''}}+A_{v^{'''}}+A_{u}+A_{u^{''}}+A_{u^{'''}}}$$

$$\left[ {Ce}^{4+} \right]=\frac{A_{v}+A_{v^{''}}+A_{v^{'''}}+A_{u}+A_{u^{''}}+A_{u^{'''}}}{A_{v_{o}}+A_{v^{'}}+A_{u_{o}}+A_{u^{'}}+A_{v}+A_{v^{''}}+A_{v^{'''}}+A_{u}+A_{u^{''}}+A_{u^{'''}}}$$

For Ceria NSs:

$$\left[ {Ce}^{3+} \right]=\frac{A_{v^{'}}+A_{u^{'}}}{A_{v^{'}}+A_{u^{'}}+A_{v}+A_{v^{''}}+A_{v^{'''}}+A_{u}+A_{u^{''}}+A_{u^{'''}}}$$

$$\left[ {Ce}^{4+} \right]=\frac{A_{v}+A_{v^{''}}+A_{v^{'''}}+A_{u}+A_{u^{''}}+A_{u^{'''}}}{A_{v^{'}}+A_{u^{'}}+A_{v}+A_{v^{''}}+A_{v^{'''}}+A_{u}+A_{u^{''}}+A_{u^{'''}}}$$

where *A_i_* is the integrated area of peak “*i*”.**Supplemental Table S2.** List of qRT-PCR primers used in the study.

| **Gene** | **Species** | **Sequences for primers** | |
| --- | --- | --- | --- |
| Actin | mouse | Forward | 5′-GGC ACC ACA CCT TCT ACA ATG-3′ |
|  |  | Reverse | 5′-GGG GTG TTG AAG GTC TCA AAC-3′ |
|  | human | Forward | 5′-GTG CTA TCC CTG TAC GCC TC-3′ |
|  |  | Reverse | 5′-GGC CAT CTC TTG CTC GAA GT-3′ |
| SOD1 | mouse | Forward | 5′-CAG AAG GCA AGC GGT GAA C -3′ |
|  |  | Reverse | 5′-CAG CCT TGT GTA TTG TCC CCA TA-3′ |
|  | human | Forward | 5′-TGA AGA GAG GCA TGT TGG AGA-3′ |
|  |  | Reverse | 5′-TGC CCA AGT CAT CTG CTT TTT-3′ |
| SOD2 | mouse | Forward | 5′-CCA AGA CCT GCC TTA CGA CTA-3′ |
|  |  | Reverse | 5′-GGT GGC GTT GAG ATT GTT GA-3′ |
|  | human | Forward | 5′-GGA AGC CAT CAA ACG TGA CTT-3′ |
|  |  | Reverse | 5′-GCA GTG GAT CCT GAT TTG GAC-3′ |

**Figure S1.** (**A**) Length distribution (of 317 particles evaluated) and (**B**) diameter distribution of Ceria NRs (228 particles evaluated). (**C**) Particle size distribution of Ceria NSs (218 particles evaluated).


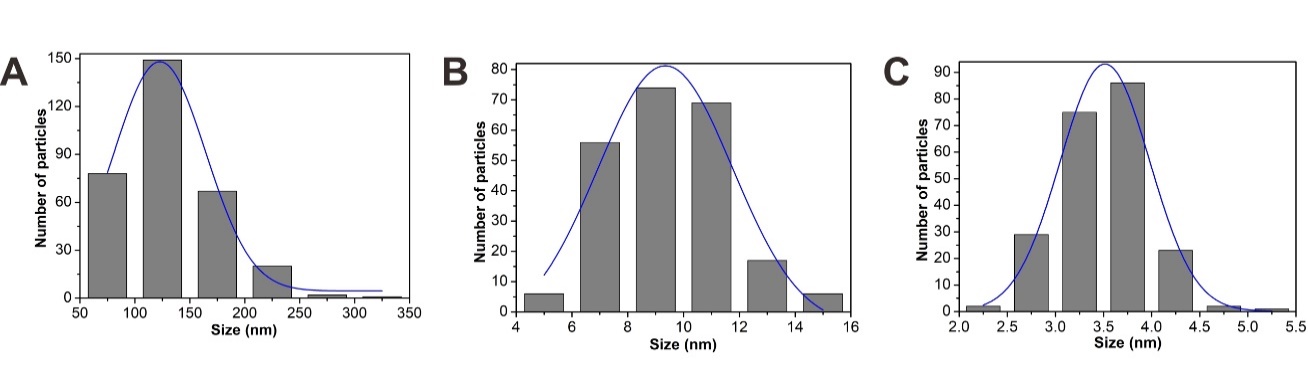


**Figure S2.** TEM image and EDX elemental mapping data of (**A**) Ceria NRs and (**B**) Ceria NSs showing cerium (in red) and oxygen (in green).


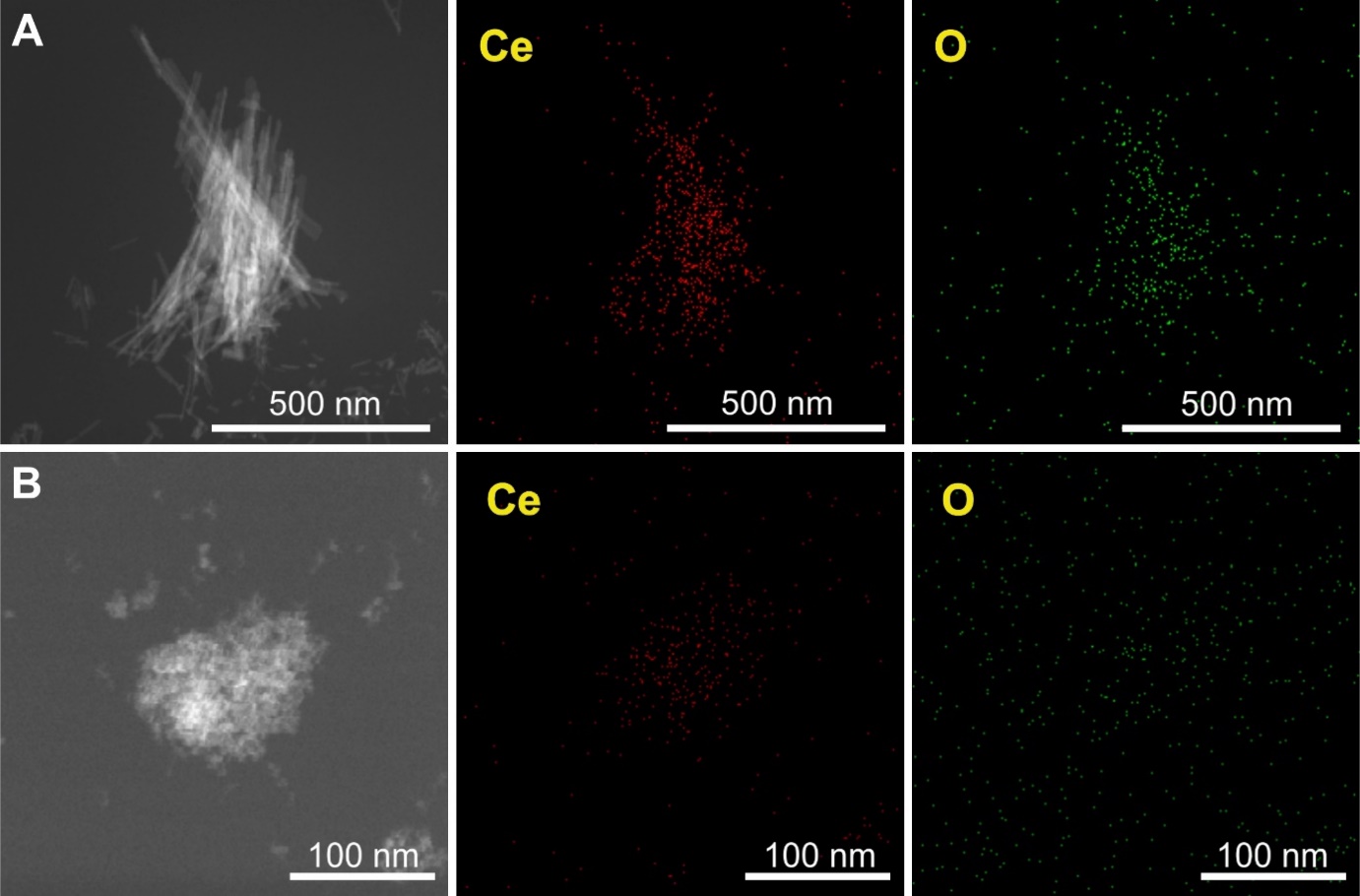


**Figure S3.** Nitrogen sorption measured at 77 K for Ceria NRs (**A**) and Ceria NSs (**B**).


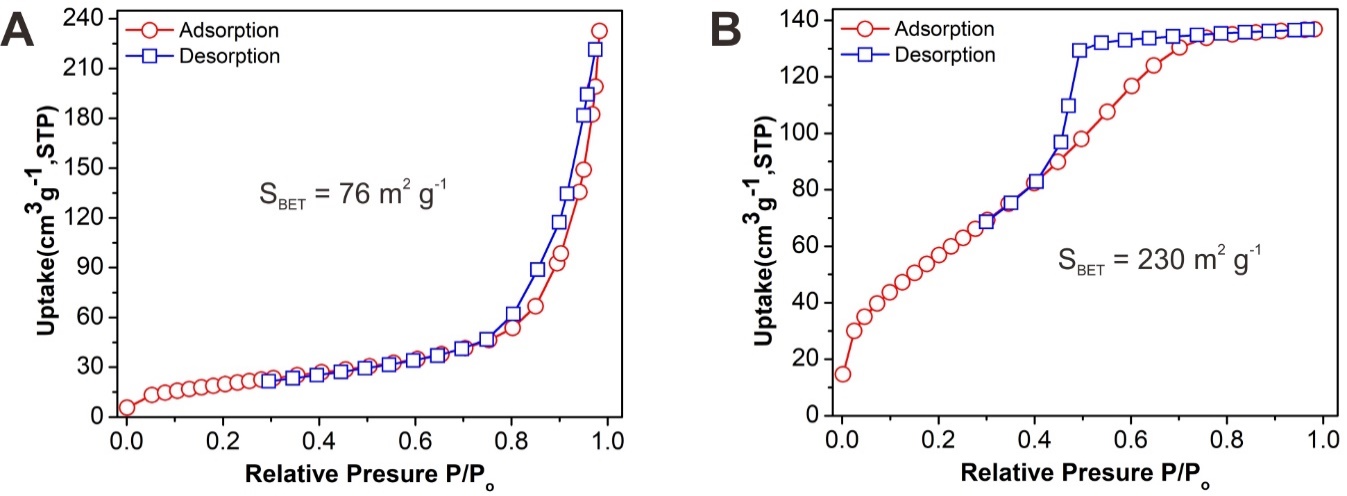


**Figure S4.** Full scans of original unprocessed western blots presented in Figure 3G. In red, the cropped area used in the main figure.


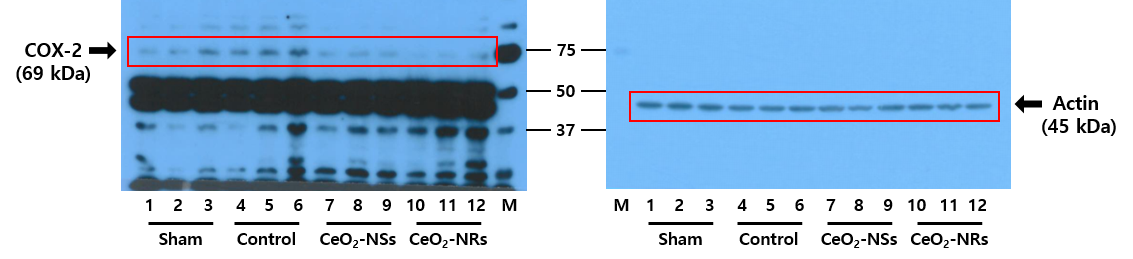


**Supplemental Methods**

**TUNEL assay**

*In vivo* apoptotic cell death was detected using the DeadEnd^TM^ Fluorometric TUNEL System (Promega, USA). The brains were rapidly resected from the perfused control (sham) and TBI-induced mice treated with or without ceria nanoparticles, embedded in optimal cutting temperature compound, and snap frozen. Serial horizontal cryosections measuring 30 μm in thickness were placed on slides. According to the manufacturer's protocol, the slide was stained with TUNEL reaction mixture and analyzed via fluorescence microscopy to detect the green fluorescence. The slide was counter-stained with 4, 6-diamidino-2-phenylindole (DAPI).

**FJB staining**

The cryosection slides were immersed in 0.06% potassium permanganate for 15 min and washed for 1 min with distilled water. Slides were stained with 0.001% Fluoro-Jade B (Histo-Chem Inc., Jefferson, AR, USA) solution for 30 min. After washing three times in distilled water for 5 min, slides were then dried at 55 ℃ for at least 35 min in the dark and coverslipped with D.P.X (Sigma-Aldrich Co., USA). The stained brain tissues were observed at 450–490 nm using a fluorescence microscope (Carl Zeiss, Germany).

**qRT-PCR**

Total RNA was isolated from brain tissues with easy-BLUE (Invitrogen). The cDNA was synthesized using the Maxime RT PreMix kit (iNtRON Biotechnoloty, Korea) in accordance with the manufacturer’s instructions. PCR reactions were performed with the 2X Rotor-Gene SYBR Green PCR Master Mix (Qiagen, USA) in the Rotor-Gene Q (Qiagen, USA). The primers are listed in Table S2. PCR was performed for 45 cycles under the following conditions: 94°C for 15 s, 55°C for 30 s, and 70°C for 30 s.

**Brain water content**

The wet weight of brain tissue was determined, followed by drying in the oven at 100 °C for 3 h to obtain the dry weight. Cerebral edema was estimated using the following formula: % Water content = 100*(wet weight - dry weight)/wet weight. Six independent experiments were conducted per group (n=6). The experimental groups were divided into three groups of six mice (C57BL/6 J-male) each: mTBI, mTBI with Ceria NRs, and mTBI with Ceria NSs.

**Western blot**

Brain tissue and cells were lysed in RIPA buffer containing proteinase inhibitor K. Protein concentrations were measured using the PierceBCA Protein Assay Kit (Thermo Scientific). Equal amounts of cell extracts were loaded for western blotting analysis. The primary antibodies used in this study were anti-COX2 (sc-7877; Abcam), anti–β-actin (ab8227; Abcam).

**Novel object recognition test**

Mice were adapted in an open field arena (65 x 45 x 30 cm) for 2 days, 10 min on each day. Mice were then acclimated individually within the arena for 3 days, 10 min daily, for two identical objects (A1 and A2). After 24 hours, the mice were exposed for 10 min to a familiar object A1 and a novel object designated as B1. Three days after the induction of mTBI, the mouse activity was evaluated using the distance traveled in an open field test for 10 min between the same object A1 and a novel object named C1. Object exploration was defined as rearing of the object, sniffing it at a distance of less than 2 cm and/or touching it with the nose. Object preference was measured once when the distance between the nose in mice and the object was within 2 cm. All of the animal behavior tests were recorded via video tracking system and presented using heat map tracking images (Noldus Ethovision, Leesburg, VA). The blue color represents the less-visited area and red color denotes frequently-visited area.
